# The Relevance of a Conductor Competition for the Study of Emotional Synchronization Within and Between Groups in a Natural Musical Setting

**DOI:** 10.3389/fpsyg.2019.02954

**Published:** 2020-01-17

**Authors:** Thibault Chabin, Grégory Tio, Alexandre Comte, Coralie Joucla, Damien Gabriel, Lionel Pazart

**Affiliations:** ^1^Laboratoire de Neurosciences Intégratives et Cliniques EA481, Université Bourgogne Franche-Comté, Besançon, France; ^2^Centre Hospitalier Universitaire de Besançon, Centre d’Investigation Clinique INSERM CIC 1431, Besançon, France; ^3^Plateforme de Neuroimagerie Fonctionnelle et Neurostimulation - Neuraxess, Besançon, France

**Keywords:** emotional synchronization, EEG, hyperscanning methods, music, conductor competition

## Abstract

Group emotional dynamics are a central concern in the study of human interaction and communication. To study group emotions, the social context of a musical event in natural conditions may overcome several limits of laboratory experiments and could provide a suitable framework. This study aimed to evaluate if cultural events such as a conductor competition could welcome scientific research for the study of group emotional sharing. We led an observational study, which suggests that in this particular context, public, musicians and jury would agree to participate and to wear neurophysiological and physiological devices to monitor their emotional state during the competition. Self-administrated scales showed that, in the context of a musical competition, members of the public felt strong musical emotions such as music chills. Our results suggest that such a specific competition design is a suitable experimental model to lead an experiment under ecological conditions to effectively investigate collective emotional synchronization. In the future, with the implementation of an acquisition system recording synchronous neurophysiological data for a large group of participants, we may be able to highlight mechanisms involved in emotional synchronization in a natural musical setting.

## Introduction

Most people listen to music for its emotional effect (Nieminen et al., 2012) and research has shown the pleasurable effect of music (Grewe et al., 2007; Salimpoor et al., 2009, 2011). The social connection with other people and being connected to the performers are the main factors that motivate people to attend live performances and enjoy them (Burland and Pitts, 2014; Leante, 2016; Swarbrick et al., 2019). Music is a form of cultural communication (McCormick, 2009). According to the model of the Share Affective Motion Experience (Molnar-Szakacs et al., 2011), which claims music as “a series of intentional, expressive motor acts, recruiting similar neural networks in both agent and listener” (Overy, 2012: 66), music can enhance emotions and create a sense of social bonding (Overy, 2012; Chanda and Levitin, 2013).

Considering research that has investigated the effect of music listening in groups, some points out that context (laboratory or natural conditions) and the social environment (being alone or with other people) could greatly influence the experience of listening to music (Lamont, 2009; Liljeström et al., 2013). Being in the context of a concert in the presence of other people can enhance the listening experience by eliciting at least a rhythmic synchronization when people move together (Desmet et al., 2010). Live conditions provide listeners with the strongest musical experiences (Lamont, 2011), with a greater effect on physiological variables as heart rate (Shoda et al., 2016). However, contradictory results have emerged from several studies. On the one hand, more “pleasure enjoyment” was experienced during music listening with a close friend or a partner versus alone (Juslin et al., 2008), and people who listened to music alone were less likely to experience intense emotions compared to people listening with a close friend or a partner (Liljeström et al., 2013). On the other hand, an intra-individual variability has been demonstrated. Some people were more likely to experience chills when listening to music alone, whereas others experienced more when they were with others in daily life situations (Nusbaum et al., 2013). Furthermore, in a laboratory setting, the social context negatively affected the emotional experience: the experience of chills, and thus emotional arousal, was lower in groups (Egermann et al., 2011). The social context, particularly in this laboratory setting experiment, influenced participants’ concentration, altering the emotional arousal (Egermann et al., 2011; Koehler and Broughton, 2017). However, these studies in laboratory or natural contexts with dyads or groups involved major differences in implementation.

Ecological conditions may be of great importance when considering the precise mechanisms of human interactions (Acquadro et al., 2016). An ecological context should clarify and help generalize previous laboratory findings (Egermann et al., 2013), surpassing dyadic interactions (Tarr et al., 2014) and going beyond some of the experimental laboratory limits in the study of group emotions. Collective music performances are a suitable tool through which to study interactions in ecological conditions (D’Ausilio et al., 2015). Thus, to study interpersonal synchronization and the mechanisms involved in social interactions, it is possible to simulate research protocols that are as realistic as possible whilst maintaining ecological validity. Ecological experiments in the musical context have already measured head movement synchronization by simulating a live concert in a living lab (Swarbrick et al., 2019), or measured the heart rate of a few people among a large audience listening to a piano performance in an auditorium (Shoda et al., 2016). Otherwise, when natural settings are not reproducible in ecological conditions, it is possible to benefit from the specific design arising from a natural situation.

By definition natural experiments are studies conducted in a natural everyday life context seeking to measure variations in natural processing without the manipulation of variables (Salkind, 2010). To set up a study in natural conditions, the setting should be stable over time and should include natural exogenous variations without any interventions from the experimenters (Meyer, 1995; Craig et al., 2012). The drawback of such natural conditions is that the selection of participants, the adherence of subjects with the research, the implementation of subjective measures, the adaptability of the paradigm to environmental conditions and so on need to be evaluated precisely before implementing a study. In this respect, we hypothesized that the specific context of a conductor competition would bring an advantageous framework to study the spread of emotions and emotional synchronization within and between groups (music performers and listeners) in natural conditions. In this context, both cerebral activity, in terms of natural activation of the autonomic nervous system, and subjective feeling could be recorded among the different participants (orchestra performers, jury, candidates, and public) during the competition.

In the specific context of the conductors’ competition, the same orchestra performing the same musical pieces in the same concert hall in front of the same public offers favorable live setting conditions. The spread of emotions can be assessed within and between groups of performers and listeners. Contrary to laboratory settings, the musical material is ecological in this context. Direct reports of emotional responses from participants and neurophysiological recordings should give objective and subjective measures for the evaluation of groups’ emotional synchronization. In this context, any intervention of the experimenter would influence the responses. Furthermore, the natural response of the participants should be controlled at a physiological level. Music chills can be defined as a strong transient emotional phenomenon triggered by specific musical features and involving reactions of the brain and body in the form of goose bumps and thrills, and which can provoke intense pleasure (Goldstein, 1980; Salimpoor et al., 2011). Chills present the advantage of being identified both by physiological measures and directly reported by the participant (Grewe et al., 2009a, b, 2011). Blood and Zatorre (2001) suggest that pleasantness and emotional intensity must increase to a certain level before the occurrence of a chill. Musical chills that are the climax of an increase in emotion (Grewe et al., 2009a) should be a reliable indicator of a strong emotional state. They can be generated in a social situation such as a concert, movie theater, or other gathering where people are involved in the same action and focused on the same target (Schoeller, 2015). In that respect, music chills could indirectly be an indicator of common emotions between several participants when chills are experienced at the same time. Using this observational approach should allow experimenters to record a set of data on different interacting populations, wherein synchronization can be evaluated in a natural way, without no intervention required, throughout the competition.

Volpe et al. (2016) insisted on acquiring large data samples in valid ecological conditions using multimodal tools to assess synchronization. Measuring subjective feelings and peripheral arousal during live concerts provides a great opportunity to study the influence of other people on emotional responses to music (Egermann et al., 2013). The new ambulatory monitoring techniques of physiological recording could highlight numerous as yet unknown fundamental mechanisms and datasets related to information in the field of emotion research, especially in studies in ecological conditions (Wilhelm and Grossman, 2010; Shoval et al., 2018). Some musical research has already been coordinated in both passive and active conditions. A number of studies have shown the effect of listening to music in terms of the emotional response using physiological recording, considering both cardiac and electrodermal activities (Craig, 2005; Grewe et al., 2011; Mori and Iwanaga, 2014; Colver and El-Alayli, 2016). Moreover, the development of electroencephalographic (EEG) hyperscanning methods allows researchers to record EEG data on several participants simultaneously, providing an appropriate tool for the study of brain processes involved in social interactions (Dumas et al., 2010; Acquadro et al., 2016; Toppi et al., 2016; Balconi and Vanutelli, 2017).

In line with this approach, we led an observational study that is a first step in the implementation of natural experiments using neurophysiological sensors in natural conditions. The overall goal was to assess the appropriateness of using a conductor competition to study emotional synchronization within and between groups (music performers and listeners) in natural conditions. Three dimensions were assessed during this observational study: the acceptability for listeners (public, jury) and performers (musicians, conductors) of wearing different sensors during the competition; the emotional sensitivity of the public in a such context; and the suitability of competition settings to replicate an experimental design in natural conditions.

## Materials and Methods

### Ethics Statement

The research met the local ethical rules provided by French law on non-invasive protocols involving healthy participants, and was classified as a psychological observational study outside the Jardé law (Article R1121-1 of the French Law Code of Public Health amended by decree n° 2017-884 of May 2017). It was submitted to the Ethics Committee CPP Est II, which exempted the study from the full ethics review process. Each participant was informed of the observational nature of the study. Participants freely filled out questionnaires and gave their implicit consent by returning the questionnaire. The questionnaires were anonymous, and no participants can be identified. Participants who were interviewed gave their explicit oral consent before answering the investigator’s questions.

### Presentation of the International Competition for Young Conductors

The 55th Besançon International Competition for Young Conductors in France is a musical event based on the musical direction of various styles and various ensembles (two pianos, opera, symphonic orchestra, etc.). From the pre-selection to the final phase, every candidate has to perform the musical direction of the same musical extracts. Our study concerns only the final phases. The final rounds of the competition lasted several days and were composed of two sessions per day, the first during the afternoon and the second in the evening. We named data collection sessions according to the day (Monday: Mon; Tuesday: Tue; Wednesday: Wed) and according to the time of day: afternoon session [1] or evening session [2]. We collected data in five sessions: during the 1/8 elimination round (Mon1 and Mon2), the 1/4 final round (Tue1 and Tue2), and the first semi-final round (Wed1). To identify the candidates engaged in the competition, letters from A to T were assigned to each of the 20 conductors.

The 1/8 elimination round took place at the Grand Kursaal on Monday, 11 September 2017. Ten candidates during the first session (Mon1) and ten candidates during the second session (Mon2) conducted the Orchestre Victor Hugo de Besançon Franche-Comté using three extracts of approximately 10 min from the musical piece “Appalachian’s Spring Orchestral Suite” by Aaron Copland. The 1/4 elimination round took place at the Ledoux Theater on Tuesday, 12 September. Six candidates during the first session (Tue1) and six candidates during the second (Tue2) conducted the Orchestre Victor Hugo de Besançon Franche-Comté using three extracts of approximately 20 min from “Carmen” by Georges Bizet. The first semi-final round took place at the Grand Kursaal and included six candidates, all of whom participated in both sessions. First, they conducted the Orchestre National de Lyon using two extracts lasting approximately 25 to 30 min from the ‘Concerto No.2 for Piano and Orchestra in F minor, Op.21’ by Frédéric Chopin (Soloist: Philippe Cassard) (Wed1). For further details on the last rounds of the competition, see Supplementary Data 1.

### Location

The final rounds took place in two concert halls in Besançon: the Grand Kursaal and the Ledoux Theater. The Grand Kursaal is a 480 m^2^ concert hall containing two balconies and a great stage; it is able to accommodate approximately 800 people. The Ledoux Theater is a classic theater composed of a stage and a pit for the orchestra; it is able to accommodate 1,000 persons on three levels.

### Hypothesis and Objectives

We hypothesized that the specific context of this conductor competition would provide an advantageous framework to measure the emotional synchronization within and between groups (music performers and listeners) in natural conditions using neurophysiological devices.

To test this hypothesis, three requirements must be addressed:

1. *Acceptability of further study*: Will participants agree to wear neurophysiological sensors during the competition? (Feasibility for recruitment).
2. *Public’s emotional sensitivity and public profile*: Will public members be sensitive to musical emotions and to music chills? Will they be representatives of the general public of classical concert? (External validity).
3. *Suitability of competition setting*: Will the extracts from each music piece be performed by each conductor for a minimal duration of 1 min/30 s? (Reproducibility of similar conditions) Could some confounding factors (e.g., types of music piece, orchestra, architecture of the concert hall) be managed to avoid measurement bias? Will a significant number of conductors produce music chills in the public? (At least 30% should experience chills).

### Study Design

In this observational study, different activities were conducted to answer our three main requirements.

1. Acceptability for a Further Study
We conducted face to face interviews to assess the willingness of some public members, jury members, musicians, and conductors to wear sensors (EEG headsets and wristbands recording physiological data) during the conductor competition.
2. Public’s Emotional Sensitivity and Public Profile
We distributed self-reported questionnaires to evaluate personality profiles and public emotions in the specific context of the competition, involving a lot of repetitions of the same short extract of music pieces.
3. Suitability of Competition Setting
We did observations of the competition setting to ensure the right reproducibility of each candidate’s performance for further statistical analysis.

### Population

To assess the acceptability of the research protocol, the different groups were jury members (*n* = 7), public members (around 300 to 400 per session), musicians (*n* = 45), and conductors (*n* = 20) engaged in the competition. See Table 1 for demographic information about the participants involved. Interviewees were selected based on their availability during the breaks (before, during and after the performance). To test the public’s musical emotional sensitivity, the only eligibility criterion considered for the distribution of all questionnaires and for collecting opinions was to be aged over 18 years. Participation was on a voluntary basis.

**TABLE 1:** Public sample by session.

**Session**	**Questionnaires distributed**	**Questionnaires returned (%)**	**Males/Females (%)**	**Mean age (SD)**
Mon1	90	45 (50)	34/66	60.9 (15)
Mon2	71	23 (32.3)	24/76	54.5 (16.4)
Tue1	120	21 (17.5)	19/81	67.5 (8.4)
Tue2	150	39 (26)	25/75	50.7 (16.6)
Wed1	150	38 (25.3)	30/70	63.5 (14)

### Variables, Data Sources and Measurement

#### Acceptability of Further Study

A sample of the four groups (orchestra performers, jury, candidates, and public) was questioned orally about the acceptability of both the type of mobile EEG headset and the tools that could be used in such conditions (three different mobile devices were presented in pictures), along with whether they would agree to participate in a similar study about emotions in the next edition of the competition. The interviews were conducted individually by TC and LP, mainly during the intermissions, detailing our aim, the setting for the study and the different requirements involved in participation. Because of competition conditions, interviews were conducted directly in the concert rooms and personal information about age and gender were not collected during oral interviews. The only criterion was to be aged over 18 years, with no further discrimination. The mobile tools presented were EEG wireless headsets – namely Emotiv Insight, Emotiv Epoc+ and Imec’s wireless EEG headset – and connected wristbands such as Jawbone UP3 and Fitbit. Oral interviews were conducted individually with candidates, musicians and members of the jury during intermissions. The interviews were conducted with the musicians and with the conductors during the 1/8 final round (Mon1, Mon2). They were conducted with members of the public during the five data collection sessions (Mon1, Mon2, Tue1, Tue2, and Wed1).

#### Public’s Emotional Sensitivity: A Public Profile

Self-reported questionnaires were used to ascertain the public profile. Each questionnaire gave only the age and sex of the responder. Questionnaires were distributed at each entrance to the concert hall before the performance. Participants freely filled out and returned the questionnaire at the end of the session.

Many psychology studies have pointed out the positive link between the personality trait of openness to experience and sensitivity to aesthetic/music chills (Grewe et al., 2007; McCrae, 2007; Nusbaum and Silvia, 2011a; Silvia and Nusbaum, 2011; Sumpf et al., 2015; Colver and El-Alayli, 2016). We chose to assess the emotional sensitivity of responders and their sensitivity to music chills with a French adaptation of the Aesthetic Experiences Scale in Music or AES-M (Nusbaum and Silvia, 2011b; Sachs et al., 2016). Finally, we used an adaptation of the AES-M by candidate (emotional scale by candidate, ESC) to make comparisons between each candidate and each performance. Since musical emotional sensitivity could be linked with the personality profile, we also used the French adaptation of the Ten Item Personality Inventory (TIPI), extracted from the Big Five personality domains (BFI) (Gosling et al., 2003). Using the personality profile increased the internal validity of our data. Finally, the classification of the French Orchestra Association (AFO, Association Française des Orchestres, 2014) was used to compare public from the conductor competition with public from classical concert. All these scales are presented in brief below and in Supplementary Appendices 1–3.

##### Ten Item Personality Inventory

The TIPI (see Supplementary Appendix 1) is composed of ten items, five of which are opposed to the other five. This allows the researchers to calculate five personality trait scores: extraversion, agreeableness, conscientiousness, emotional stability, and openness to experience. Each personality trait score is calculated by averaging the score of one item with the reverse score of its opposite item on a seven-point Likert scale. The reverse score corresponds to the reverse rating of the score (for example, if the score is one, the reverse score will be seven; if the score is two, the reverse score will be six, etc.). The item “extraverted, enthusiastic” is opposed to the item “reserved, quiet,” giving a score for the extraversion personality trait. The item ‘critical, quarrelsome’ is opposed to the item “sympathetic, warm,” giving a score for the agreeableness personality trait. The item “dependable, self-disciplined” is opposed to the item “disorganized, careless,” giving a score for the conscientiousness personality trait. The item “anxious, easily upset” is opposed to the item “calm, emotionally stable,” giving a score for the emotional stability personality trait. Finally, the item ‘open to new experiences, complex’ is opposed to the item “conventional, uncreative,” giving a score for the openness to experience personality trait.

##### Aesthetic experience scale in music

Based on Nusbaum and Silvia’s previous work (Nusbaum and Silvia, 2011b) (see Supplementary Appendix 2), four factors were calculated for the AES-M from ten items: the chill factor, the touched factor, the absorption factor and the global factor. The chill factor is composed of the items “Feel chills down your spine,” “Get goose bumps” and “Feel like your hair is standing on end,” giving scores out of three. The absorption factor is composed of the items “Feel absorbed and immersed,” “Completely lose track of time,” “Feel like you’re somewhere else,” “Feel detached from your surroundings” and “Feel a sense of awe and wonder,” giving scores out of five. The touched factor is composed of the items “Feel touched” and “Feel like crying,” giving scores out of two. The score for a factor indicates how many items of this factor had a response greater than or equal to four on a seven-point Likert scales (corresponding to “sometimes”). The global factor score out of ten was calculated for each participant by adding the three factor scores.

##### Emotional scale by candidate

This scale (see Supplementary Appendix 3) tests whether people can feel different emotions or musical chills according to the conductor. The scale by candidate is composed of the items extracted from the AES-M that participants checked off for each candidate. This allows the researchers to calculate four factors similar to those of the AES-M. The chill factor gives scores out of three, the absorption factor gives scores out of five, and the touched factor gives scores out of two. A global factor score out of ten was calculated for each participant by adding the three factor scores. The absence of a response was considered as no feeling for the item by a candidate.

##### French Orchestra Association (AFO) Classification

During the semi-final, people were asked to define their auditor profile by choosing between the profiles extracted from a classification of the French Orchestra Association (Association Française des Orchestres, 2014). These categories were “classical music lover”: first, interested in classical music and then, sharing a moment in a beautiful place; “curious music lover”: focused on a musical experience, interested in discovering new musical works and searching for new emotions aroused by musical work; “sociable”: concert experience is a pleasurable moment shared with friends and family in the musical emotion register; “occasional layman”: infrequent musical and concert practice, came with family or friend and enjoyed the spectacular dimension of the event; “distanced amateur”: concerts are an uncommon opportunity and not a usual practice.

##### Course of the observational study

The schedule of different activities is presented in Table 2.

**TABLE 2:** Schedule of activities.

**Activities**	**Mon1 First 1/8 elimination round (10 conductors)**	**Mon 2 Second 1/8 elimination round (10 conductors)**	**Tue 1 First 1/4 round (6 conductors)**	**Tue 2 Second 1/4 round (6 conductors)**	**Wed 1 First semi-final (6 conductors)**
Interviews	X	X	X	X	
AES-M	X	X	X		
ESC				X	X
TIPI	X	X	X	X	
AFO					X
Direct observation of setting	X	X	X	X	X

### Suitability of Competition Settings

Finally, direct observations of the concert hall and of the competition process were performed by noting the timing of the performance and by analyzing the musical work to determine whether the competition was adapted to neurophysiological recordings.

### Statistical Analysis

Statistical analysis only covers self-reported questionnaires to describe the public profile.

A Shapiro–Wilk test was used to check that the data as a whole followed a normal distribution. Descriptive statistics were analyzed for the age, gender and size of the sample to determine the characteristics of the public who responded to the questionnaires during each session (presented in Table 3). For all statistical tests, the *p*-value was fixed with α = 0.05 and analysis involved a Bonferroni’s correction based on the multiplicity of the tests. The comparison between sessions for TIPI scores was analyzed for all participants using analysis of variance (ANOVA) for extraversion and emotion traits, and a Kruskal–Wallis test for agreeableness, consciousness and openness to experience traits (correction of the *p*-value threshold to *p* = 0.01).

**TABLE 3:** Acceptability of further study.

**Categories**	**Size of the population**	**Size of the sample**	** Agreement, number (%)**
Jury	7	5	5 (100)
Public	400	36	34 (94)
Musicians	45	15	15 (100)
Conductors	20	13	9 (70)
Total		69	63 (91)

#### Emotional Sensitivity According to Candidates

A non-parametric repeated measures ANOVA GLIMMIX procedure under SAS was applied for each ESC factor for Tue2 and Wed1 conductors (correction of the *p*-value threshold to *p* = 0.0125) to compare the ratings for each of the conductors (random effect), followed by a *post hoc* contrast analysis with a *p*-value correction (*p*-value threshold: 0.0033). Effect size were calculated with Kendall coefficient of concordance (W). For the ESC, a descriptive analysis of the chills felt (indicating the number of people who reported at least one item of the chill factor) and the number of people who reported chills for other candidates are presented in Table 4.

**TABLE 4:** The number of people reporting feeling chills, by candidate (Tue2 and Wed1).

			**Tue2 *N* = 39**			
**Candidate**	**S**	**E**	**O**	**I**	**L**	**P**

Felt chills, *n* (%)	7 (17.9)	9 (23.1)	5 (12.8)	3 (7.7)	9 (23.1)	2 (5.1)
Felt chills for other candidates, *n*	6	6	5	3	5	2

			**Wed1 *N* = 37**			

**Candidate**	**O**	**C**	**R**	**H**	**I**	**F**

Felt chills, *n* (%)	5 (13.5)	7 (18.9)	9 (24.3)	5 (13.5)	5 (13.5)	4 (10.8)
Felt chills for other candidates, *n*	3	6	4	3	2	3

#### AFO Classification

Chi^2^ tests were applied to compare the results of the AFO classification in our study and in the AFO survey. Effect size were calculated with Cramer’s V.

## Results

### Acceptability of Further Study

Sixty-nine individuals were questioned orally during the whole competition. Table 3 shows the sample size of the different groups (members of the jury, members of the public, members of the orchestra and conductors engaged in the competition) that agreed to wear sensors for a future study.

Among the 20 conductors, 13 were interviewed, of whom eight gave their agreement; one more agreed as long as devices were worn by all candidates. Among the four conductors who refused to wear sensors, three of them indicated the possible discomfort of the EEG headset as they already wear a microphone, and the fourth cited the distraction the sensor could cause. All of them agreed to participate in the study if the research protocol was included in the competition without wearing sensors. In summary, 91% of people interviewed agreed to wear sensors to participate in a future study during the conductor competition.

### Public’s Emotional Sensitivity and Public Profile

Five hundred and eighty one questionnaires were distributed during five sessions of the competition, of which 166 were returned, giving a response rate of 28.6%. See Table 1 for details.

Regarding the TIPI scales, 128 questionnaires were returned. There were no significant differences in sessions for any items of the TIPI.

Regarding the AES-M, 89 questionnaires were returned. Descriptive results indicate that the factor “touched” was rated greater than or equal to four for 85 participants during the competition. This suggests that 95.6% of the participants indicated that they felt “touched” during the competition. In the same way, 70 participants (78.7%) indicated being “absorbed” and 29 (32.6%) indicated feeling chills. Finally, the global factor indicated that there were 26 participants (29.2%) who rated at least one item of each factor greater than or equal to four.

Regarding the ESC, 77 questionnaires were returned and 76 were analyzed (for the analysis, a letter from A to T was assigned to each candidate). For the Tue2 session, public reports showed significant differences between candidates for the global factor [*F*(5,190) = 5.13; *p* < 0.0001; *W* = 0.12585] and *post hoc* analysis revealed differences between candidates S (*M* = 1.54; SD = 1.5) vs. L (*M* = 2.64; SD = 1.8; *p* < 0.001), I (*M* = 1.41; SD = 1.43) vs. L (*p* < 0.0001) and L vs. P (*M* = 1.26; SD = 1.33; *p* < 0.0001). Candidate L had a higher global factor rating than the other candidates. For the Tue2 session, there were also significant differences for the absorption factor [*F*(5,190) = 3.65; *p* < 0.0125; *W* = 0.07608]. The *post hoc* analysis revealed differences between candidates S (*M* = 1.13; SD = 1.06) vs. L (*M* = 2; SD = 1.52; *p* < 0.01), I (*M* = 1.33; SD = 1.36) vs. L (*p* < 0.001) and L vs. P (*M* = 1.08; SD = 1.20; *p* < 0.001). For more details, see Supplementary Appendix 4. Candidate L had a higher global factor rating than the other candidates. There were no significant differences for chill factor between candidates [*F*(5,190) = 2.04; *p* = 0.069; *W* = 0.06533) or for the touched factor [*F*(5,190) = 2.25; *p* = 0.0464; *W* = 0.07506]. For Wed1, there were no significant differences between candidates for any factor of the AES-M [touched factor *F*(5,180) = 5,180; *p* = 0.696; *W* = 0.01679; absorption factor *F*(5,180) = 1.51; *p* = 0.182; *W* = 0.04666; chill factor *F*(5,180) = 0.70; *p* = 0.626; *W* = 0.0288] or global factor [*F*(5,180) = 1.41; *p* = 0.222; *W* = 0.0486]. Table 4 presents the number of people reporting chills (at least one item of the chill factor) by candidate for both Tue2 and Wed1. The phrase “I felt chills for other candidates” indicates the number of people who also indicated experiencing chills for other candidates.

During the Tue2 session, 15 participants (38.4%) indicated feeling chills, and during the Wed1 session, 14 participants (37.8%) indicated feeling chills. During the whole competition (Mon1, Mon2, Tue1, Tue2, and Wed1), 58 participants (34.9%) indicated feeling chills.

Regarding the AFO classification, 32 members of the public completed the self-reported AFO categories. The results indicate that there are significantly more “curious music lovers” compared to the AFO public survey (53.1% vs. 15.7%; *p* < 0.0001; *V* = 0.00542); there are significantly fewer “sociable” respondents in our study than in the AFO survey (9.4% vs. 29.6%; *p* < 0.05; *V* = −0.0234) and there are significantly fewer “distanced amateurs” in our study than in the AFO survey (3% vs. 20.9%; *p* < 0.05; *V* = −0.0231). There are no significant differences between the “occasional layman” and “classical music lover” profiles.

### Suitability of Competition Settings

For Mon1 and Mon2, the candidates’ performances were timed; they had precisely 10 min. During each performance, they conducted two extracts for every session, with at least one common extract. For the first semi-final round (Wed1), the candidates had, on average, 23.8 min (SD = 2.7) to perform. They all conducted the second movement during this round. Table 5 indicates that, except for candidate F, they all had approximately 5 min for free rehearsals, and all rehearsed the second movement with the orchestra. During all the elimination rounds, a real time video of a front view of each conductor’s performances was projected onto a large screen. In this way, the audience and the jury could follow the gestures, skills and facial expressions of the candidates.

**TABLE 5:** Movements, rehearsal and timing for each candidate’s performance during the first semi-final round (Wed1) for the second movement.

**Order (candidates)**	**Duration of the whole performance (mins)**	**Conducting time for the 2nd movement (mins)**	**Rehearsal work of the 2nd movement (mins)**
First (O)	29	18	From measure 27 (6)
Second (C)	23	15	From measure 27 (6)
Third (R)	23	12	From measure 27 (4)
Fourth (H)	23	15	From measure 42 (5)
Fifth (I)	24	13	From measure 37 (5)
Sixth (F)	17	8	Discussion with the pianist and rehearsal (4)

The segmented part of the competition should enable suitable monitoring of physiological recordings and an analysis of little portions of signal. We identified some suitable spaces to prepare participants, for the positioning of the computers, and for the monitoring of the recordings. For the theater, recordings on musicians would be more complex because of the reduced space in the pit, but was still feasible.

## Discussion

The results of this study show the suitability of using a conductor competition as a testbed to study emotional synchronization within and between groups (music performers and listeners) in a natural setting. The conductor competition provides a natural experimental design: during a session, the same orchestra plays the same extract many times in the same place in front of the same public. Thus, every conductor conveyed a different musical interpretation and different emotional content in a randomized running order that constituted natural variation.

### Would People Agree to Wear the Sensors During the Competition?

Interestingly, 91% of the interviewees in the different groups agreed to participate by wearing mobile devices. All the people from the jury and the musicians that were interviewed agreed. Only two people from the public were inconsistent with our approach. This result suggest that our research is well accepted by the public, jury and musicians. Even though the organizers of the competition gave their consent and more than 71% of the interviewed conductors agreed, there were four conductors that refused to wear sensors during the competition. For conductors, it might be complicated to implement neurophysiological recordings in terms of the management of technical constraints and not disturb their performance. They may have been afraid of the devices. Nevertheless, all conductors agreed to participate in a study if the research protocol was acceptable for all candidates and was included in the competition rules. However, these results should be carefully considered: agreements in principle are more easily given when people are not directly engaged in the experiment thereafter.

### Did the Public Experience Emotions During the Competition?

More than 95% of the participants indicated feeling touched during the competition, according to AES-M questionnaires. Our analysis of the general emotions reveals that a sufficient proportion of the public (29.2%) were able to feel emotions during the first few sessions of the competition. These results also confirm that in the particular context of the competition, even if the musical pieces are repeated and performed with completely different interpretations than the public’s expectations, people can feel emotions. Furthermore, a high percentage of respondents (34.9%) reported feeling chills during the competition. This result strengthens the validity of our approach to the investigation of strong musical emotions. Our evaluation of the public’s emotional sensitivity was positive for the first sessions, so we used the ESC on Tue2 and Wed1 to make deeper comparisons between each candidate and each performance. We used the ESC especially when the number of candidates was reduced to six and thus comparisons were easier to make. We confirmed that some candidates conveyed greater emotions to the public than other candidates. In a future natural experiment, conductors conveying greater emotions should also elicit greater emotional synchronization between the musicians and the audience. Less emotional performances could be used as control. During a single performance, some candidates succeeded in triggering chills in almost a quarter of our respondent sample. This suggests that future experiments in natural conditions might highlight an emotional synchronization between participants during a conductor’s performance. In sum, our public was sensitive to musical emotion in the natural context of the competition. Furthermore, people that are not sensitive to musical emotions should be a necessary control group for the measurement of emotional synchronization in the future natural experiment.

Music chills area well-established marker of musical emotional peaks and a quite large majority of humans should be sensitive to music chills – as high as 86%, according to Panksepp (1995) – while some (anhedonics) are not. Music chills do not occur systematically, and many factors are implicated (personality profile, conditions of listening, musical pieces, social atmosphere, temperature of the room, and so on). Our study shows that music chills occurred in a third of the listeners that completed the self-reports under the specific conditions of the contest. The importance of this rate is certainly debatable, but the sample of respondents was not selected on their predisposition to feel chills. This criterion should probably be introduced in a subsequent research study. The current observation simply shows that chills occurred quite frequently despite the conditions of the conductor competition, with short musical excerpts and frequent interruptions of the orchestra. Considering the future naturalistic experiment, this result indicates that an adequate sample can be selected from the public at the competition.

### Could Competition Settings Facilitate Experiments?

Considering the competition setting, our observations of the concert halls’ characteristics show that neurophysiological recording can be set up in these natural conditions. During all the sessions, each candidate conducted at least one common extract over a sufficient time period and had at least 20 min of rehearsal (except for one candidate). It would allow statistical comparisons of future neurophysiological data to be made. Thus, neurophysiological recordings could be set up to give enough time to obtain comparable data. As they are not directly engaged in the competition as jury members or musicians, we can hypothesize that members of the public would be cognitively unfettered and thus more able to concentrate on their emotional state for the continuous reporting. With their stationary position allowing for easy control of technical constraints, the public appear to be most able to endure neurophysiological recording. Our population was homogeneous between the sessions: in the different sessions, no significant differences were highlighted for the rating of each item of the TIPI. Since personality profiles are similar between each session, neurophysiological recordings could be implemented in all phases of the competition. This is a strong argument for the feasibility of a future study.

The natural environment lets the public have the flow of natural auditory and visual information matching with the context (Williamon et al., 2014). Moreover, the issue of a conductor or a music competition, impossible to reproduce in simulated contexts, reinforces the immersive experiment and keeps motivation and attention throughout the sessions. This context allows the public to judge every performance. Considering the musical material, the stimuli are ecological and not simulated. To broadcast repeated stimuli many times for neurophysiological recordings in a laboratory experiment is inappropriate. Furthermore, in the condition of a classical concert, classical orchestras’ rehearsals are impossible to reproduce, while numerous repetitions are necessary to make comparisons and thus obtain neurophysiological data with a valid statistical analysis. A conductor or a music competition overcomes this difficulty. A stationary instrumental ensemble, in particular a classical one, performing the same musical score several times in the same conditions limits the uncertainty and limits the influence of different natural variables on the neurophysiological data collection. This specific design sets a fixed frame with natural control of musical stimuli in natural conditions. This is essential in studies using music and EEG, since the different features of music (pitch, tone, beat, rhythm) can influence brainwaves in a different way. The conductors will influence the whole orchestra in different ways, conveying different emotional interpretations of musical pieces and producing the aesthetic quality of the ensemble (Boerner and von Streit, 2007). Finally, the participants’ responses are a reliable indicator of emotion that needs to be recorded. The natural feedback of participants should not be influenced by any element of the experiment other than the performance itself.

### Limitations

Some biases might be implied. Some members of the public might not have fully understood all the terms of the TIPI scale. This kind of questionnaire is most often used in a general context, so our case study context was not totally appropriate. Moreover, though our response rate was reasonable, the sample size remains too small to draw comprehensive conclusions. Our population is slightly different from the general public for classical music performance: in the context of the competition, our sample has significantly more “curious music lovers” and fewer “sociable” respondents than the AFO public survey. These characteristics limit the external validity of our results. However the “curious music lovers,” defined as particularly interested in the music offered in the context and searching for new musical works, experiences and emotions, are of particular interest in relation to our emotional approach. Furthermore, as mentioned, some people should not have the same opinion about our approach if that implicate to be directly engaged in the competition instead of giving a simple agreement in principle. Because of the competition timing, we could not gather the opinions of all respondents regarding the wearable devices: this meant we were unable to correlate the agreement of the participants and scales. Finally, since one aim of the study was to evaluate the feasibility of neurophysiological recordings in natural conditions, we did not record audio and video motion that would be appropriate for study of the orchestra and public (Swarbrick et al., 2019). Audio and video motion, combined with neurophysiological recordings, could offer a unique approach in the understanding of emotional synchronization and should be included in future research.

### Perspectives

These results are part of a larger work investigating emotional synchronization in groups in a natural framework. Our project gathers many facets of non-verbal human interactions (emotional, physical, aesthetic and sensorimotor) and several kinds of interaction in terms of directional categorization: observation condition, turn-based interaction and continuous interaction (Acquadro et al., 2016) in a particular context. Including both the audience and the musicians in a multidirectional interaction increases the ecological validity; unfortunately, though, it also increases the uncertainty of the ongoing measures (D’Ausilio et al., 2015). However, the recording of EEG hyperscanning and physiological data synchronized with the direct behavioral response of multiple participants on a smartphone through different groups will be a huge source of inter-related data, giving complex and precious information about human interactions in passive and active musical contexts. Some studies using EEG hyperscanning have investigated the synchronization between musicians playing in groups (Babiloni et al., 2006; Lindenberger et al., 2009; Müller et al., 2012, 2013). The technological progress provided by recently developed portable EEG equipment combined with increasingly powerful artifact detection methods (Stone et al., 2018) facilitates realistic experiments, especially those studying several musicians simultaneously (Babiloni et al., 2011, 2012; Müller et al., 2012, 2013, 2018). As previously emphasized, classical musicians performing a musical score in a relatively stationary position allows researchers to record EEG data with a high signal-to-noise ratio (Babiloni et al., 2011; D’Ausilio et al., 2015), as well as a good physiological recording. However, though a few studies have recorded EEG and physiological data during musical performances (Babiloni et al., 2011; Ichihashi et al., 2012; Müller et al., 2012, 2013, 2018), one of the main experimental limits is still the control of motor execution, greatly disrupting the signal. Musicians are often instructed to limit their musical execution movements as much as possible. Considering this difficulty, musicians’ neurophysiological recordings have to be tested and managed before the competition to ensure good signal and minimal noise.

In a future natural experiment, we aim to recruit regulars and people interested primarily in the competition and not in the scientific study. This experiment in natural conditions may highlight as yet unknown cerebral and physiological mechanisms between an active group of musicians and a passive public. We suggest that good synchronization between musicians might have an influence on public emotions. The higher the emotional synchronization in the musician’s group, the more public members should be synchronized. Such a hypothesis will be assessed by objective and subjective measures.

## Conclusion

These results confirm that this specific conductor competition is a suitable model to investigate collective emotions in natural conditions using neurophysiological devices. Almost all of the people interviewed agreed in principle to wear a sensor; the competition’s emotional content is appropriate for the study of collective emotions; and the settings of the competition fit our neurophysiological approach. The competition design is similar to a natural standard experimental design. Thus, the conductors conveying different emotional performances constitute the intervention, with a randomized running order. Particular attention must be paid to participants’ musical emotional sensitivity. This competition context – particularly an audience gathering in a concert hall – may highlight a number of undiscovered mechanisms involved in emotional synchronization.

## Data Availability Statement

The datasets generated for this study are available on request to the corresponding author.

## Ethics Statement

This research work has been approved as conforming to the French law by an ethics committee. It was classified as a psychology observational study outside of the Jardé law (Article R1121-1 of the French Law Code of Public Health amended by decree n°2017-884 of May 2017). Each participant was informed of the observational nature of the study and gave their explicit consent by answering the investigator’s questions. The questionnaires were anonymous and do not allow the identification of any participants.

## Author Contributions

LP: conceptualization. TC, AC, DG, CJ, and LP: data curation. TC and GT: formal analysis. TC and LP: writing – original draft. TC, AC, DG, LP, and GT: writing – review and editing.

## Conflict of Interest

The authors declare that the research was conducted in the absence of any commercial or financial relationships that could be construed as a potential conflict of interest.
