# Supplementary material for: The Relevance of a Conductor Competition for the Study of Emotional Synchronization Within and Between Groups in a Natural Musical Setting
Source: Front Psychol. 2020 Jan 17;10:2954. doi: 10.3389/fpsyg.2019.02954 (PMC6979053; doi:10.3389/fpsyg.2019.02954)
Supplement: Supplementary file 1 [file Data_Sheet_1.docx]

**Supplementary data**

Supplementary 1: competition design

Appendix 1: Ten Item Personality Inventory (TIPI)

Appendix 2: Aesthetic Experience Scale in Music (AES-M)

Appendix 3: Emotional Scale by Candidate (ESC)

Appendix 4: Table A

**Supplementary 1**

Competition design

Pre-selection round

Pre-selection consisted of a direct musical conduction and took place in April and May 2017 in Beijing, China (35 candidates), Berlin, Germany (115 candidates), Montreal, Canada (30 candidates) and Besançon, France (90 candidates), selecting 20 candidates for the final rounds which took place in Besançon in September 2017. For the pre-selection steps, participants had to conduct and lead a rehearsal of a reduction of two musical pieces for two pianos (**Ludwig van Beethoven**, *Symphony n°1 in C major* (opus 21) and **Hector Berlioz**, *Romeo and Juliette*/Symphonic extracts.)

Lasts rounds of the competition.

For the second round of the semi-final, the same six conductor conducted the Orchestre National de Lyon accompanied by the Aedes Vocal Ensemble on an oratorio; *B minor mass BWV 232* from **Jean-Sebastien Bach**. The final round took place on Saturday, September 16^th^; three candidates conducted the Orchestre National de Lyon on symphonic pieces; first ‘*Le Jardin étoilé*’ from **Philippe Hersant**, then two extracts of *Nocturnes* (*Nuages; Fêtes*) from **Claude Debussy** and finally, the overture of ‘*The Bat*’ from **Johann Strauss**. Finally, the jury awarded ‘the grand prize for conducting’ to the winner.

**Appendix 1**

**Ten Item Personality Inventory (TIPI)**

A French translation provided by Dr. Erica Carlisle (ECarlisle@rmsg.com) of Rosetta Marketing Strategies Group, Princeton, NJ was used. According to Dr. Carlisle, ‘The translations were done by a very large and reputable global market research company and double checked by a second set of native speakers’. *

**Usually, how do you feel about yourself?**

|  | Never | Almost never | Infrequently | Sometimes | Frequently | | | Almost always | Always |
| --- | --- | --- | --- | --- | --- | --- | --- | --- | --- |
| Anxious, easily upset | 🞎 | 🞎 | 🞎 | 🞎 | | 🞎 | 🞎 | | 🞎 |
| Reserved, quiet | 🞎 | 🞎 | 🞎 | 🞎 | | 🞎 | 🞎 | | 🞎 |
| Extraverted, enthusiastic | 🞎 | 🞎 | 🞎 | 🞎 | | 🞎 | 🞎 | | 🞎 |
| Critical, quarrelsome | 🞎 | 🞎 | 🞎 | 🞎 | | 🞎 | 🞎 | | 🞎 |
| Dependable, self-disciplined | 🞎 | 🞎 | 🞎 | 🞎 | | 🞎 | 🞎 | | 🞎 |
| Open to new experiences, complex | 🞎 | 🞎 | 🞎 | 🞎 | | 🞎 | 🞎 | | 🞎 |
| Sympathetic, warm | 🞎 | 🞎 | 🞎 | 🞎 | | 🞎 | 🞎 | | 🞎 |
| Disorganised, careless | 🞎 | 🞎 | 🞎 | 🞎 | | 🞎 | 🞎 | | 🞎 |
| Calm, emotionally stable | 🞎 | 🞎 | 🞎 | 🞎 | | 🞎 | 🞎 | | 🞎 |
| Conventional, uncreative | 🞎 | 🞎 | 🞎 | 🞎 | | 🞎 | 🞎 | | 🞎 |

**Appendix 2**

**Aesthetic Experience Scale in Music.**

Please rate each item of the questionnaire from ‘Never’ to ‘Always’ after listening to all candidates.

| **When you listened to candidates you…** | Never | Almost never | Infrequently | Sometimes | Frequently | Almost always | Always |
| --- | --- | --- | --- | --- | --- | --- | --- |
| Feel absorbed and immersed | 🞎 | 🞎 | 🞎 | 🞎 | 🞎 | 🞎 | 🞎 |
| Completely lose track of time | 🞎 | 🞎 | 🞎 | 🞎 | 🞎 | 🞎 | 🞎 |
| Feel chills down your spine | 🞎 | 🞎 | 🞎 | 🞎 | 🞎 | 🞎 | 🞎 |
| Get goose bumps | 🞎 | 🞎 | 🞎 | 🞎 | 🞎 | 🞎 | 🞎 |
| Feel like you’re somewhere else | 🞎 | 🞎 | 🞎 | 🞎 | 🞎 | 🞎 | 🞎 |
| Feel like your hair is standing on end | 🞎 | 🞎 | 🞎 | 🞎 | 🞎 | 🞎 | 🞎 |
| Feel like crying | 🞎 | 🞎 | 🞎 | 🞎 | 🞎 | 🞎 | 🞎 |
| Feel touched | 🞎 | 🞎 | 🞎 | 🞎 | 🞎 | 🞎 | 🞎 |
| Feel detached from your  surroundings | 🞎 | 🞎 | 🞎 | 🞎 | 🞎 | 🞎 | 🞎 |
| Feel a sense of awe and wonder | 🞎 | 🞎 | 🞎 | 🞎 | 🞎 | 🞎 | 🞎 |

**Appendix 3**

**Emotional Scale by Candidate**

Please check items of the questionnaire after listening to each candidate if you experience it.

| When you listened to candidates n° | Candidate 1 | Candidate 2 | Candidate 3 | Candidate 4 | Candidate 5 | Candidate 6 |
| --- | --- | --- | --- | --- | --- | --- |
| Feel absorbed and immersed | 🞎 | 🞎 | 🞎 | 🞎 | 🞎 | 🞎 |
| Completely lose track of time | 🞎 | 🞎 | 🞎 | 🞎 | 🞎 | 🞎 |
| Feel chills down your spine | 🞎 | 🞎 | 🞎 | 🞎 | 🞎 | 🞎 |
| Get goose bumps | 🞎 | 🞎 | 🞎 | 🞎 | 🞎 | 🞎 |
| Feel like you’re somewhere else | 🞎 | 🞎 | 🞎 | 🞎 | 🞎 | 🞎 |
| Feel like your hair is standing on end | 🞎 | 🞎 | 🞎 | 🞎 | 🞎 | 🞎 |
| Feel like crying | 🞎 | 🞎 | 🞎 | 🞎 | 🞎 | 🞎 |
| Feel touched | 🞎 | 🞎 | 🞎 | 🞎 | 🞎 | 🞎 |
| Feel detached from your surroundings | 🞎 | 🞎 | 🞎 | 🞎 | 🞎 | 🞎 |
| Feel a sense of awe and wonder | 🞎 | 🞎 | 🞎 | 🞎 | 🞎 | 🞎 |

**Appendix 4**

**Table A. Mean, Standard deviation for each factor of each candidate of Tue2 session.**

|  | **Candidates** | | | | | |
| --- | --- | --- | --- | --- | --- | --- |
|  | **S** | **E** | **O** | **I** | **L** | **P** |
| **Absorption factor**  **Mean (*SD*)** | 1.13*(1.06)** | 1.44*(1.14)* | 1.33*(1.36)* | 1.15*(1.16)** | 2*(1.52)* | 1.08*(1.20)** |
| **Touched factor**  **Mean (*SD*)** | 0.15*(0.43)* | 0.26*(0.5)* | 0.31*(0.52)* | 0.18*(0.45)* | 0.36*(0.49)* | 0.10*(0.31)* |
| **Chill factor**  **Mean (*SD*)** | 0.26*(0.64)* | 0.33*(0.74)* | 0.18*(0.51)* | 0.08*(0.27)* | 0.28*(0.6)* | 0.08*(0.35)* |
| **Global factor**  **Mean (*SD*)** | 1.54*(1.5)** | 2.03*(1.56)* | 1.82*(1.55)* | 1.41*(1.43)** | 2.64*(1.80)* | 1.26(*1.33)** |

**Significant difference compared to candidate L.*
